# Supplementary material for: Quantifying the contribution of subject and group factors in brain activation
Source: Cereb Cortex. 2023 Sep 27;33(22):11092–101. doi: 10.1093/cercor/bhad348 (PMC10646690; doi:10.1093/cercor/bhad348)
Supplement: Supplementry_Material_bhad348 [file supplementry_material_bhad348.zip › Supplementry_Material_bhad348.docx]

Quantifying the contribution of subject and group factors in brain activation

Johan Nakuci^*^, Jiwon Yeon, Kai Xue, Ji-Hyun Kim, Sung-Phil Kim and Dobromir Rahnev

Corresponding Author: Johan Nakuci

Email: [jnakuci@gmail.com](mailto:jnakuci@gmail.com)

**This file includes:**

Figures S1 to S8

**Fig. S1. Trial-level analysis maps of voxel activation consistency across subjects**. A) Task-based activation. B) RT-based activation. C) Confidence-based activation. All maps exhibited strong areas of consistency. Analysis was conducted on fMRI data smoothed with 5, 10, and 20 mm FWHM kernels. The 10 mm results are the same as in the main manuscript and are shown here for comparative purposes. Again, similar results are obtained for different levels of smoothing.

**Fig. S2. Trial-level maps of the distribution of the top-10% most activated voxels**. A) Task-based activation. B) RT-based activation. C) Confidence-based activation. All maps exhibited strong areas of consistency compared. Analysis was conducted on fMRI data smoothed with 5, 10, and 20 mm FWHM kernels. The 10 mm results are the same as in the main manuscript and are shown here for comparative purposes. Again, similar results are obtained for different levels of smoothing.

**Fig S3. Comparing the Full, Subject-Only, and Group-Only models**. Sample within-subject reliability and subject-to-group similarity from the simulation using the Full, Subject-Only, and Group-Only factors in the simulation for (A) task-, (B) RT-, and (C) confidence-based activations. The full simulation model used subject-, group-, and noise-factors. The Subject-Only simulation model used subject and noise factors. The Group-Only simulation model used group and noise factors. (D-F) Model performance. The within-subject reliability and subject-to-group similarity values estimated in 25 simulations, (D) the mean-squared error (MSE), (E) AIC, and (F) BIC were estimated by comparing the within-subject reliability and subject-to-group similarity from the simulation with the empirical values. The Full model outperformed both the Subject-Only and Group-Only models. Error bars show SEM. *** p < 0.001.

**Fig. S4. Within-subject reliability and subject-to-group similarity for analyses conducted at the block level.** Within-subject reliability and subject-to-group similarity values of the whole-brain maps produced by the (A) task-, (B) RT-, and (C) confidence-based analyses. We fit a general linear model (GLM) that allowed us to estimate the beta values for each voxel in the brain. For the block-analyses, the model consisted of regressors for each individual block (block onset was set to the beginning of fixation on the first trials and block offset was set to the confidence response of the last trial in the block), inter-block rest periods, as well as linear and squared regressors for six head movement (three translation and three rotation), five tissue-related regressors (gray matter, white matter, cerebrospinal fluid, soft tissues, and air and background), and a constant term per run. Two behavior-based analyses compared the beta values for blocks with faster- vs. slower-than-median average reaction times (RT) and higher- vs. lower-than-median average confidence. Within-subject reliability and subject-to-group similarity of the whole-brain maps produced by the task, RT, and confidence analyses was examined in the same manner as for the trial level analysis. The fMRI data were spatially smoothed with 5 mm, 10 mm, or 20 mm full width half maximum (FWHM) Gaussian kernel. As can be observed, very similar results are obtained for different levels of smoothing, indicating that the results obtained are likely due to large-scale rather than small-grained differences in the maps. Error bars show SEM.

**Fig. S5. Block-level maps of voxel activation consistency across subjects**. A) Task-based activation. B) RT-based activation. C) Confidence-based activation. Task-based activations exhibited strong areas of consistency, but both the RT and confidence maps showed much weaker consistency across subjects. Analysis was conducted on fMRI data smoothed with 5, 10, and 20 mm FWHM kernels. Again, similar results are obtained for different levels of smoothing.

**Fig. S6. Block-level maps of the distribution of the top-10% most activated voxels**. A) Task-based activation. B) RT-based activation. C) Confidence-based activation. Task-based activations exhibited strong areas of consistency, but both the RT and confidence maps showed much weaker consistency across subjects. Analysis was conducted on fMRI data smoothed with 5, 10, and 20 mm FWHM kernels. Again, similar results are obtained for different levels of smoothing.

**Figure S7. Block-level model weights, ratios, and proportions.** A) Model weights. Subject- and group-level weights obtained from fitting the model separately to the data with each smoothing level. B) Weight ratios. Relative weights of the subject-level and corresponding group-level factors for each smoothing level. C) Factor proportions. The relative weight of subject, group, and noise factors contributing to the activation on an individual block. Subject and group factors reflect the summed task, RT, and confidence weights.

**Fig S8. Comparing the Full, Subject-Only, and Group-Only models for block-level analysis**. Sample within-subject reliability and subject-to-group similarity from the simulation using the Full, Subject-Only, and Group-Only factors in the simulation for (A) task-, (B) RT-, and (C) confidence-based activations. The full simulation model used subject-, group-, and noise-factors. The Subject-Only simulation model used subject and noise factors. The Group-Only simulation model used group and noise factors. (D-F) Model performance. The within-subject reliability and subject-to-group similarity values estimated in 25 simulations, (D) the mean-squared error (MSE), (E) AIC, and (F) BIC were estimated by comparing the within-subject reliability and subject-to-group similarity from the simulation with the empirical values. The Full model outperformed both the Subject-Only and Group-Only models. Error bars show SEM. *** p < 0.001.
